# Presence Of Left Ventricular Non Compaction In Hypertrophic Cardiomyopathy Is Associated With Arrhythmia

**DOI:** 10.1186/1532-429X-18-S1-P298

**Published:** 2016-01-27

**Authors:** Ganesh Kumar Gnanappa, Preeti Choudhary, Linda Lin, Chi Jen Hsu, Caroline Medi, Stuart Grieve, David Celermajer, Chris Semsarian, Raj Puranik

**Affiliations:** 1 Paediatric cardiology, Children Hospital at Westmead, Kingsford, NSW Australia; 2 Cardiovascular magnetic resonance Sydney, Sydney, NSW Australia; 3 Royal Prince Alfred Hospital, Sydney, NSW Australia; 4 The University of Sydney, Sydney, NSW Australia; 5 Cardiology, Black Town Hospital, Sydney, NSW Australia

## Background

Cardiac magnetic resonance (CMR) imaging allows accurate assessment of ventricular compaction. We aimed to determine whether left ventricular non-compaction (LVNC) is related to increased incidence of arrhythmia in adults with Hypertrophic cardiomyopathy (HCM).

## Methods

58 consecutive HCM patients referred for CMR between 2008-2014 were recruited. Only patients with intermediate ventricular thickness of 15-29 mm were included. Patients with apical HCM, severe LV outflow tract obstruction (resting gradient >50 mmHg) and significant loading conditions, such as aortic stenosis or hypertension, were excluded. LVNC was diagnosed as per Petersen's criteria.

## Results

66% of the patients were male, mean age 52 ± 18 years, mean wall thickness was 19 ± 4 mm. Amongst the 9 patients with LVNC, 5 had VT/VF, 2 had SVT and 2 had syncope. Patients with LVNC had a significantly higher prevalence of ventricular arrhythmia than those without LVNC (56% vs 18%, p = 0.03), with a relative risk of 3.0 (95% CI 1.3 - 6.9). LV septal thickness (18.4 ± 5.6 mm vs 18.7 ± 3.5 mm, p = 0.9) and ejection fraction (70 ± 7.7 vs 70 ± 10.5%, p = 0.9) did not significantly differ between those with LVNC and those without. Presence of LV scar assessed by late gadolinium enhancement was similar between the groups (89% vs 73%, p = 0.7).

## Conclusions

Presence of LVNC may be associated with ventricular tachyarrhythmia in HCM patients and may provide a new phenotypic marker for adverse prognosis, especially in the intermediate risk group. Further studies in larger populations are required to assess its possible prognostic value.

